# Raynaud's Phenomenon: Beware of Cancers!

**DOI:** 10.7759/cureus.14009

**Published:** 2021-03-20

**Authors:** Zahida Aqodad, Houda Bachir, Habiba Alaoui, Siham Hamaz, Khalid Serraj

**Affiliations:** 1 internal Medicine and Immunohematology Cellular Therapy, Faculty of Medicine and Pharmacy, Mohamed First University, Oujda, MAR; 2 Immunohematology Cellular Therapy, Faculty of Medicine and Pharmacy, Mohamed First University, Oujda, MAR; 3 immunohematology Cellular Therapy, Faculty of Medicine and Pharmacy, Mohamed First University, Oujda, MAR; 4 Infectious Diseases, Faculty of Medicine and Pharmacy, Mohamed First University, Oujda, MAR; 5 Internal Medicine, Faculty of Medicine and Pharmacy, Mohamed First University, Oujda, MAR

**Keywords:** cancer, pathophysiology, raynaud's phenomenon

## Abstract

Raynaud's phenomenon (RP) is a frequent syndrome and often indicative of connectivitis or hemopathy. The association with solid cancers is exceptional. We report the observation of a patient hospitalized for severe RP whose etiological assessment revealed the existence of colorectal cancer. We discuss, through this clinical case, the potential physiopathological links and underline the importance of looking for underlying cancer in the face of severe, refractory to treatment, or atypical RP.

## Introduction

Raynaud's phenomenon is a transient, paroxysmal vasomotor disorder following a vasospastic response to cold or to emotional stress.

The etiological investigation consists of distinguishing the primary Raynaud's phenomenon (the most frequent) from the secondary one. As for the secondary Raynaud's, it can be indicative of many benign pathologies or fit with a paraneoplastic syndrome. We report the case of a patient who presented with Raynaud's phenomenon, which was widely explored before it could be linked to colonic adenocarcinoma.

## Case presentation

**Figure 1:**
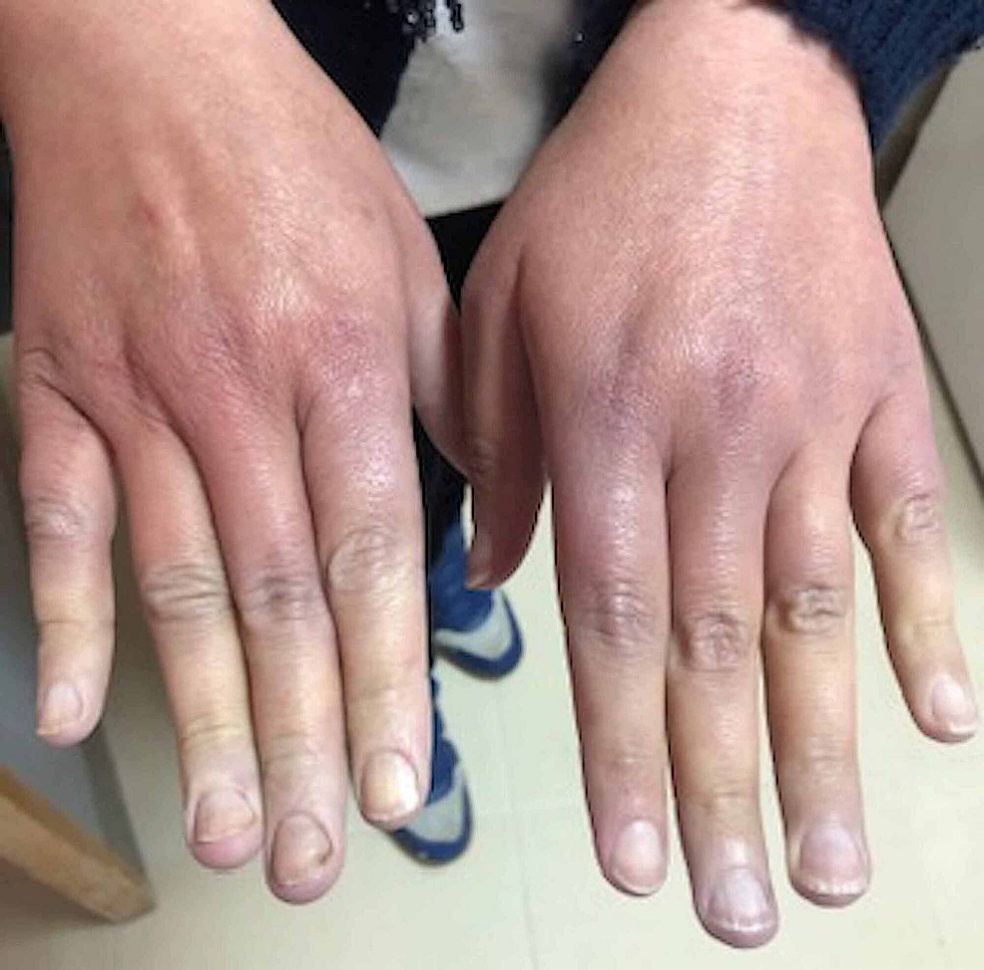
This picture indicates Raynaud's phenomenon in the hands at the time of diagnosis

A 42-year-old female patient with a history of iron deficiency anemia without smoking history or familial neoplasia consulted for inflammatory low back pain with paresthesia and pain in the fingers rebellious to simple analgesics evolving for a month before her hospitalization. The clinical examination revealed Raynaud's phenomenon of the two hands (Figure 1), which was not associated with cold, was without cutaneous involvement, arthralgia, or trophic disorder; the cardiovascular examination was normal. Blood count was normal, the anti-nuclear antibodies, anti-phospholipid antibodies, anti-neutrophil cytoplasmic antibody (ANCA), rheumatoid factor, anti-cyclic citrullinated peptides (anti-CCP), and cryoglobulinemia were negative, and capillaroscopy was not performed.

**Figure 2:**
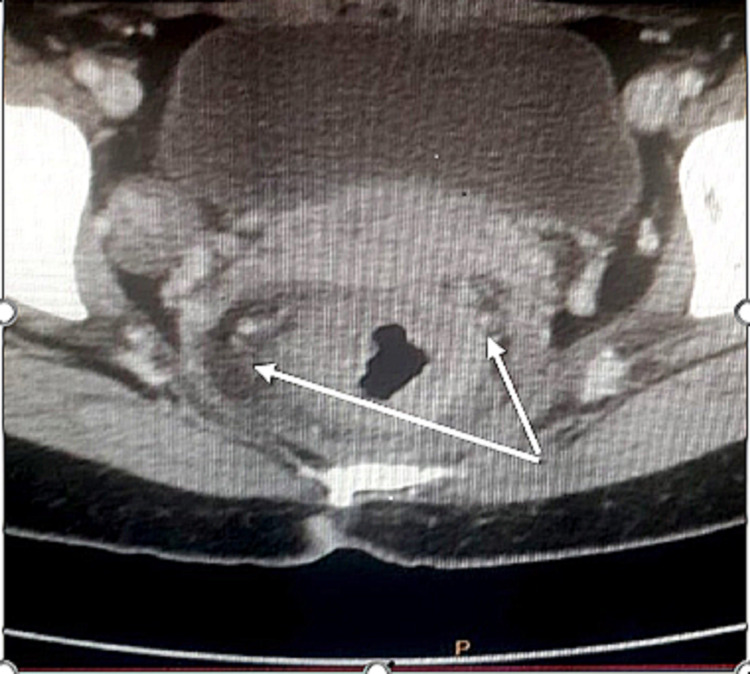
Computed tomography (CT) indicates tumor-like thickenings of the rectum

As part of the aetiological paraneoplastic assessment and given the history of an iron deficiency anemia, computed tomography revealed two tumor-like thickenings of the rectum and the sigmoid extended to the left colon, associated with deep lymph nodes (sigmoid, inferior mesenteric, and superior rectal), and nodules peritoneal carcinosis (Figure 2), and bone lesions of the spine.

**Figure 3:**
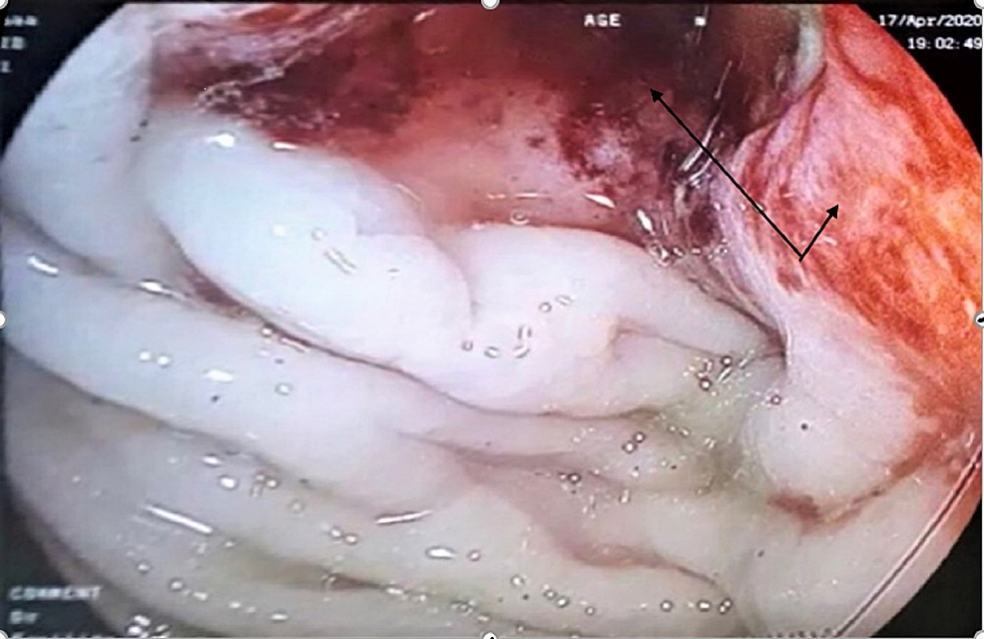
Colonoscopy image showing sigmoidal thickening

**Figure 4:**
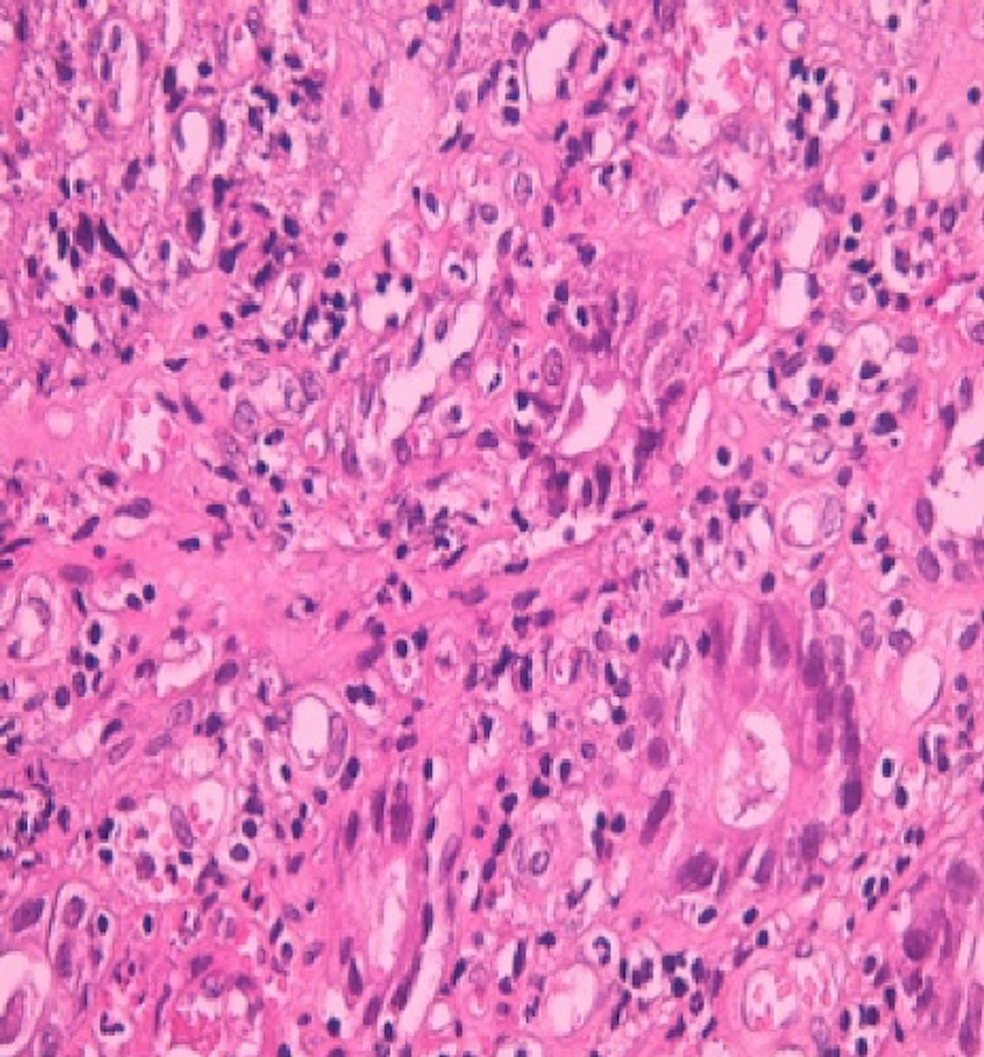
Histologic image of our patient's adenocarcinoma

The diagnostic process was completed by colonoscopy with biopsies of all lesions identified as suspect; histological study confirmed that it was a colonic adenocarcinoma (Figures 3-4) and the cancer antigen (CA) 19-9 level was up to three times the upper limit of the normal. The patient was referred to the oncology department where she received radiochemotherapy with good evolution, namely, reduction of the tumor and lymph node size, as well as remission of Raynaud's phenomenon.

## Discussion

Raynaud's phenomenon is an immunological entity affecting peripheral microcirculation, testifying to an exaggerated physiological response to cold and stress. It is classically described with a triphasic color change of the digits resulting from the succession of three phases: ischemic phase, deoxygenation phase, and reperfusion phase. The diagnosis is confirmed if these three phases are present, however, capillaroscopy, biology, and radiology have an etiological interest.

There are two types of Raynaud's phenomenon: primary and secondary. The aetiological spectrum of secondary Raynaud's phenomenon encompasses several causes; the main ones of which are vasculitis (scleroderma being the most common), toxicants or drugs, vascular disease, and neoplasms. Table 1 summarizes the main causes of secondary Raynaud's phenomenon.

**Table 1:** Main etiologies of secondary Raynaud's syndrome

Loco-regional causes	Vibration disease; Localized microtrauma; Carpal tunnel syndrome
Vasculitis	Scleroderma; Lupus; Rheumatoid arthritis; Gougerot-Sjögren
Medicines/toxicants	β-blockers; Bleomycin; Sympathomimetic Cannabis; Cocaine
Endocrine dysfunction	Hypothyroidism
Hematological disease	Cold agglutinin disease; Vaquez disease
Vascular causes	Buerger; Atheromatous arteriopathies; Thoracic outlet syndrome
Solid tumors	Breast cancers; Ovarian tumors

Paraneoplastic causes are rarely reported - 68 cases in Guigne's study and 33 cases in Schildmann's study - it is often a pulmonary, mammary, uterine, or ovarian site [1-2]. The dominant histological type is adenocarcinoma [3].

The mechanisms of the pathogenesis of Raynaud's phenomenon are not fully understood. They may be due to intravascular, neuroregulatory, or vascular disorders; the latter involving vasoconstriction phenomena occurring with the help of vasoactive agents: endothelin, angiotensin II and tyrosine kinase. Any increase in the production of these cytokines could be responsible for excessive vasoconstriction.

In cancers (mammary, colorectal, and prostate), there is an overexpression of the endothelin-1 and angiotensin II systems and their receptors. Colorectal cancer cells are endowed with a characteristic ability to activate angiotensin II, which has both a vasoconstrictive and pro-fibrotic effect [4]. They also lead to a dysregulation of tyrosine kinase, whose excessive activity is responsible for an exaggerated contractile response of the endothelial cells, which will cause vasoconstriction [5].

These physiopathological mechanisms suggest that it could have a causal link between tumor pathology, colorectal in our case, and the occurrence of Raynaud's phenomenon.

Although colorectal cancers are manifested by digestive and general symptoms, the association with Raynaud's phenomenon has only been described in one case [3].

Protective measures, prostacyclin analogs, and calcium channel blockers are the treatment of choice for idiopathic forms; otherwise, the management is that of the cause. Regarding paraneoplastic Raynauds, the regression of the phenomenon after treatment of the tumor was noted in 48% of cases [1,6].

From a diagnostic point of view, the rather unusual nature of this association makes it essential to conduct an exhaustive preliminary search for all the other causes, particularly the more classic ones of Raynaud's phenomenon, before attributing the paraneoplastic cause, which can only be retained on the exclusion of other aetiologies and especially on the improvement of clinical signs after remission of the neoplasia. In our case, all the causes of Raynaud's phenomenon were excluded and the clinical outcome was quickly favorable after the beginning of the cancer treatment.

## Conclusions

In most cases, Raynaud's phenomenon is idiopathic, especially when it occurs at a young age. However, a more exhaustive etiological investigation, including the search for neoplasia, is necessary if the phenomenon appears after the fourth decade. Our observation shows the value of researching a malignant cause before opting for the diagnosis of Raynaud's disease, especially if the initial assessment is negative and the symptoms persist despite the usual treatment.
